# Prognostic impact of postoperative complications among elderly patients with gastric cancer

**DOI:** 10.1371/journal.pone.0359100

**Published:** 2026-09-29

**Authors:** Kohei Matsuoka, Yuichiro Miki, Ryota Kawai, Masaki Nishiyama, Yuki Seki, Kenji Kuroda, Hiroaki Kasashima, Mami Yoshii, Tatsuro Tamura, Masatsune Shibutani, Takahiro Toyokawa, Ayumi Shintani, Kiyoshi Maeda

**Affiliations:** 1 Department of Gastroenterological Surgery, Osaka Metropolitan University Graduate School of Medicine, Osaka, Japan; 2 Department of Medical Statistics, Osaka Metropolitan University Graduate school of Medicine, Osaka, Japan; University of Split Faculty of Medicine: Sveuciliste u Splitu Medicinski fakultet, CROATIA

## Abstract

**Background:**

Japan’s rapidly aging population has led to an increase in elderly patients undergoing gastrectomy for gastric cancer. However, the impact of postoperative complications on survival in patients aged ≥80 years remains unclear. This study aimed to evaluate the association between postoperative complications and survival outcomes in patients aged ≥80 years with gastric cancer.

**Materials and methods:**

We retrospectively analyzed 61 patients aged ≥80 years who underwent gastrectomy with D2 lymph node dissection between 1999 and 2020. Postoperative complications were classified according to the Clavien-Dindo (CD) classification into a “no or minor complication group” (CD Grade ≤ II, including no postoperative complications) and a “major complication group” (CD Grade ≥ III), and the impact of postoperative complications on survival outcomes were assessed. A subgroup analysis excluding macroscopic type 4 was also performed.

**Results:**

Postoperative complications occurred in 24 patients (39.3%), of which 8 (13.1%) experienced major complications. Patients in the major complication group had a significantly longer postoperative hospital stay, while other baseline characteristics were similar between groups. Kaplan–Meier analysis showed a trend toward poorer OS in the major complication group (P = 0.063), and recurrence-free survival (RFS) was significantly worse in this group (P = 0.004). Multivariate analysis showed that postoperative complications of CD grade ≥ III were independently associated with poorer OS (HR 2.51; 95% CI 1.01–6.24; P = 0.049). Subgroup analysis in patients without type 4 also showed significantly worse OS in the major complication group (P = 0.036).

**Conclusion:**

This study showed that postoperative complications were associated with poorer OS and RFS even in patients aged ≥80 years with gastric cancer. Future large-scale studies are needed to further clarify this association and optimize treatment strategies for this population.

## Introduction

Japan has one of the most rapidly aging populations in the world, and the number of elderly patients diagnosed with gastric cancer continues to increase. In particular, patients aged 80 years or older currently account for more than 30% of all gastric cancer cases in Japan [1]. As the population continues to age, the number of elderly patients undergoing gastrectomy is expected to continue increasing.

Gastrectomy with D2 lymph node dissection is currently regarded as the standard surgical treatment for advanced gastric cancer. In the Dutch D1D2 trial, although postoperative complications were more frequent in the D2 group in the early postoperative period, the 15-year follow-up revealed a significant reduction in gastric cancer-specific mortality and locoregional recurrence in the D2 group [2,3]. However, evidence regarding the clinical significance of D2 lymph node dissection in elderly patients remains limited. Although several retrospective studies comparing D1 and D2 lymph node dissections in this population have been reported, the results regarding survival benefits remain inconsistent [4–10]. Therefore, further investigation is necessary to clarify the role of D2 lymph node dissection in this population. Previous studies have reported that postoperative complications independently predict worse overall survival (OS) in patients with gastric cancer [11–14]. Because elderly patients often have a higher prevalence of comorbidities, decreased physiological reserve, and reduced functional capacity, they are more prone to postoperative complications [15]. In particular, severe postoperative complications have been reported more frequently in patients aged ≥80 years than in younger patients [16,17]. In clinical practice, D2 lymph node dissection is often performed in elderly gastric cancer patients with good physical condition. Nevertheless, the prognostic impact of postoperative complications after D2 lymph node dissection in elderly patients remains unclear.

Therefore, this study aimed to evaluate the relationship between postoperative complications and survival outcomes in patients aged ≥80 years who underwent gastrectomy with D2 lymph node dissection for gastric cancer, in order to provide insight to guide better clinical decision-making.

## Materials and methods

### Patients

We retrospectively identified all consecutive patients with gastric cancer who underwent gastrectomy at our institution between January 1, 1999, and December 31, 2020. Clinical data were collected from medical records. The eligibility and exclusion criteria were as follows:

#### Eligibility criteria.

- Patients diagnosed with gastric cancer.
- Age ≥ 80 years at the time of surgery.
- Patients who underwent curative-intent surgery with D2 lymph node dissection.

#### Exclusion criteria.

- cT1 disease.
- Patients who received neoadjuvant therapy.

Finally, 61 patients were included in the analysis. This retrospective study was approved by the Institutional Review Board of Osaka Metropolitan University (Approval number:2023–013), and informed consent was obtained by opt-out manner.

The following parameters were evaluated for their association with OS:

*Patient-related factors* – age, sex, body mass index (BMI), performance status (PS), comorbidities (hypertension, diabetes mellitus, cardiovascular, respiratory, or neurological diseases), tumor location, presence of esophageal or duodenal invasion, macroscopic type, histological type, clinical stage (cStage), preoperative blood tests (lymphocytes, albumin, and C-reactive protein), and nutritional indices (Onodera’s prognostic nutritional index (PNI), geriatric nutritional risk index (GNRI), neutrophil-to-lymphocyte ratio (NLR), and modified Glasgow prognostic score (mGPS)).

*Treatment-related factors* – surgical approach, procedure, operative time, estimated blood loss, combined resection, curability, and the administration of adjuvant chemotherapy.

*Postoperative factors* – postoperative complications, postoperative hospital stay, pathological stage (pStage).

Macroscopic type, cStage, and pStage were determined according to the Japanese Classification of Gastric Carcinoma, 3rd English Edition, published by the Japanese Gastric Cancer Association [18]. Histological classification of gastric cancer was performed according to the Lauren classification, and tumors were categorized as either intestinal or diffuse type [19].

### Operative procedure and follow-up

All surgical procedures, including lymph node dissection, were performed in accordance with the Japanese Gastric Cancer Treatment Guidelines [20]. The administration of postoperative chemotherapy was decided by the attending physician based on each patients’ condition. Follow-up data were obtained through outpatient clinic records and telephone interviews.

### Assessment of postoperative complications

Postoperative complications were defined as any adverse events occurring within 30 days after surgery. Complication severity was classified using the Clavien-Dindo (CD) classification system [21]. Patients were stratified into a “*no or minor complication group*” (CD Grade ≤ II, including no postoperative complications) and a “*major complication group*” (CD Grade ≥ III). For patients with multiple complications, the highest CD grade was used for analysis.

### Statistical analysis

Categorical variables were compared using the chi-square test or Fisher’s exact test, and continuous variables were compared using the Mann-Whitney U test. We compared the clinical characteristics and surgical findings between the no or minor complication group and the major complication group. OS and Recurrence-free survival (RFS) were analyzed using the Kaplan-Meier method, and group comparisons were performed with the log-rank test. Kaplan–Meier survival analyses for OS and RFS were performed for a period of up to five years after surgery. OS was defined as the time from surgery to death, and RFS was defined as the time from the date of surgery to either recurrence or death. Patients who were alive at the time of analysis were censored at the date of last confirmed survival.

The Cox proportional hazards model was used for univariate and multivariate analyses to identify prognostic factors associated with OS. In the univariate analysis, in addition to postoperative complications, we included male sex [22] and the presence of respiratory disease [23], which have been reported as prognostic factors for OS in elderly patients with gastric cancer, as well as clinically important variables such as pT, pN, pStage, cStage, macroscopic type, histological type, PNI, surgical procedure, estimated blood loss, and operative time. Continuous variables, including PNI, estimated blood loss, and operative time, were analyzed as continuous covariates in the Cox proportional hazards model. Hazard ratios were calculated per 1-point decrease in PNI, per 100-mL increase in estimated blood loss, and per 30-minute increase in operative time. Given the limited number of events (32 deaths), the number of variables that could be included in the multivariate analysis was restricted to avoid overfitting. Therefore, the multivariate analysis was performed using two variables: pStage and postoperative complications. pStage was included because it is an established prognostic factor in gastric cancer and was considered clinically important for adjusting for potential confounding when evaluating the association between postoperative complications and OS. To assess potential overfitting, internal validation was performed using 2,000 bootstrap resamples with the *validate* function in the *rms* package, and optimism-corrected slopes were calculated [24].

All statistical analyses were performed using EZR (Saitama Medical Center, Jichi Medical University, Saitama, Japan), a graphical user interface for R (The R Foundation for Statistical Computing, Vienna, Austria) that has been modified for medical statistics. A *p*-value <0.05 was considered statistically significant. All statistical tests were two-sided.

## Results

### Patient characteristics

A total of 61 patients were included in this study, of whom 24 (39.3%) experienced postoperative complications. Among these, eight (13.1%) patients experienced major complications (CD Grade ≥ III) and were categorized into the “*major complication group*,” while the remaining 53 (86.9%) patients, including those with no or minor complications (CD Grade ≤ II), were classified as the “*no or minor complication group*”. Among the patients who met the eligibility criteria, none received neoadjuvant therapy, including chemotherapy or radiotherapy. The postoperative hospital stay was significantly longer in the major complication group than in the no or minor complication group (median 33 days vs. 14 days, P = 0.011). Although the difference was not statistically significant, adjuvant chemotherapy was administered in a higher proportion of patients in the no or minor complication group than in the major complication group (47.2% vs. 12.5%). Among patients who received adjuvant chemotherapy, none in the major complication group completed the planned treatment (0/1), whereas 24.0% (6/25) completed treatment in the no or minor complication group (S1a Table). Changes in adjuvant chemotherapy regimens during the study period are summarized in S1b Table. No significant differences were observed between the two groups regarding other patient factors, treatment factors, or postoperative factors (Table 1).

**Table 1:** Comparison of the clinical characteristics between the no or minor complication group and the major complication group.

	CD grade ≤ II	CD grade ≥ III	*P*-value
	(n = 53)	(n = 8)	
*Basic characteristics*			
Age (years)	82 (80-88)	81 (80-84)	0.363
Sex			
Male	31 (58.5%)	6 (75.0%)	0.462
Female	22 (41.5%)	2 (25.0%)	
BMI (kg/m²)	20.2 (14.3-28.5)	20.0 (17.1-25.0)	0.991
PS			
0	26 (49.1%)	2 (25.0%)	0.373
1	23 (43.4%)	6 (75.0%)	
2	4 (7.5%)	0	
Comorbidities			
Hypertension	22 (41.5%)	4 (50.0%)	0.713
Diabetes mellitus	8 (15.1%)	0	0.581
Cardiovascular disease	7 (13.2%)	1 (12.5%)	1
Respiratory disease	8 (15.1%)	0	0.581
Neurological disease	7 (13.2%)	1 (12.5%)	1
*Tumor-related characteristics*			
Tumor Location			
L	27 (50.9%)	3 (37.5%)	0.743
M	10 (18.9%)	2 (25.0%)	
U	13 (24.5%)	3 (37.5%)	
EGJ	3 (5.7%)	0	
Esophageal invasion	9 (17.0%)	0	0.591
Duodenal invasion	3 (5.7%)	0	1
Macroscopic type			
Type4	7 (13.2%)	1 (12.5%)	1
Others	46 (86.8%)	7 (87.5%)	
Histological type			
Intestinal type	32 (60.4%)	4 (50.0%)	0.706
Diffuse type	21 (39.6%)	4 (50.0%)	
cT			
2	14 (26.4%)	2 (25.0%)	1
3	26 (49.1%)	4 (50.0%)	
4	13 (24.5%)	2 (25.0%)	
cN (0/+)			
0	27 (50.9%)	2 (25.0%)	0.260
+	26 (49.1%)	6 (75.0%)	
*Pre-operation blood test*			
Lymphocyte (x10^3^/µL)	1461 (485-3524)	1650 (1100-4004)	0.550
Hb (g/dL)	11.6 (6.1-16.5)	11.6 (9.0-13.2)	0.508
Alb (g/dL)	3.6 (2.0-4.4)	3.6 (2.7-4.4)	0.789
CRP (mg/dL)	0.20 (0.01-5.64)	0.15 (0.05-0.98)	0.929
Preoperative nutritional status			
PNI	43.4 (25.6-54.8)	42.6 (39.9-53.8)	0.628
GNRI	92.3 (65.8-104.2)	87.6 (75.0-107.2)	0.765
NLR	2.5 (0.9-29.7)	2.6 (1.1-3.3)	0.612
mGPS			
0	44 (83.0%)	8 (100%)	1
1	2 (3.8%)	0	
2	5 (9.4%)	0	
*Surgical data*			
Surgical approach			
Laparotomy	49 (92.5%)	8 (100%)	1
Laparoscopic	4 (7.6%)	0	
Operative procedure			
TG	24 (45.3%)	5 (62.5%)	0.460
DG	29 (54.7%)	3 (37.5%)	
Operative time (min)	211 (106-554)	233 (184-300)	0.310
Estimated blood loss (mL)	260 (10-4550)	375 (100-1700)	0.227
Combined resection			
+	34 (64.2%)	5 (62.5%)	1
-	19 (35.8%)	3 (37.5%)	
Residual tumors			
R0	49 (92.5%)	7 (87.5%)	0.518
R1	4 (7.6%)	1 (12.5%)	
Postoperative hospital stay (days)	14 (8-84)	33 (10-68)	0.011
Adjuvant chemotherapy	25 (47.2%)	1 (12.5%)	0.122
*Pathological factors*			
pT			
1	3 (5.7%)	1 (12.5%)	0.549
2	11 (20.8%)	1 (12.5%)	
3	15 (28.3%)	1 (12.5%)	
4	24 (45.2%)	5 (67.5%)	
pN			
0	19 (35.8%)	3 (37.5%)	0.124
1	7 (13.2%)	2 (25.0%)	
2	17 (32.1%)	0	
3	10 (18.9%)	3 (37.5%)	
pStage			
I	9 (17.0%)	1 (12.5%)	0.730
II	17 (32.1%)	3 (37.5%)	
III	24 (45.3%)	3 (37.5%)	
Ⅳ	3 (5.7%)	1 (12.5%)	

Data are presented as median (range) for continuous variables and n (%) for categorical variables.

BMI, body mass index; PS, performance status; EGJ, esophagogastric junction; cT/N, clinical T/N; Hb, hemoglobin; Alb, albumin; CRP, C-reactive protein; PNI, prognostic nutritional index; GNRI, geriatric nutritional risk index; NLR, neutrophil-to-lymphocyte ratio; mGPS, modified Glasgow prognostic score; TG, total gastrectomy; DG, distal gastrectomy; pT/N/Stage, pathological T/N/Stage.

### Kaplan-Meier survival analysis

When stratified by complication severity, the 5-year OS rate was 52.0% in the no or minor complication group (median OS: not reached) and 15.0% in the major complication group (median OS: 619 days). Although the difference was not statistically significant, there was a trend toward worse OS in the major complication group (P = 0.063) (Fig 1a). Among the non-cancer-related deaths, two patients in the major complication group died directly from postoperative complications (CD grade V) (S2 Table).

**Fig 1:**
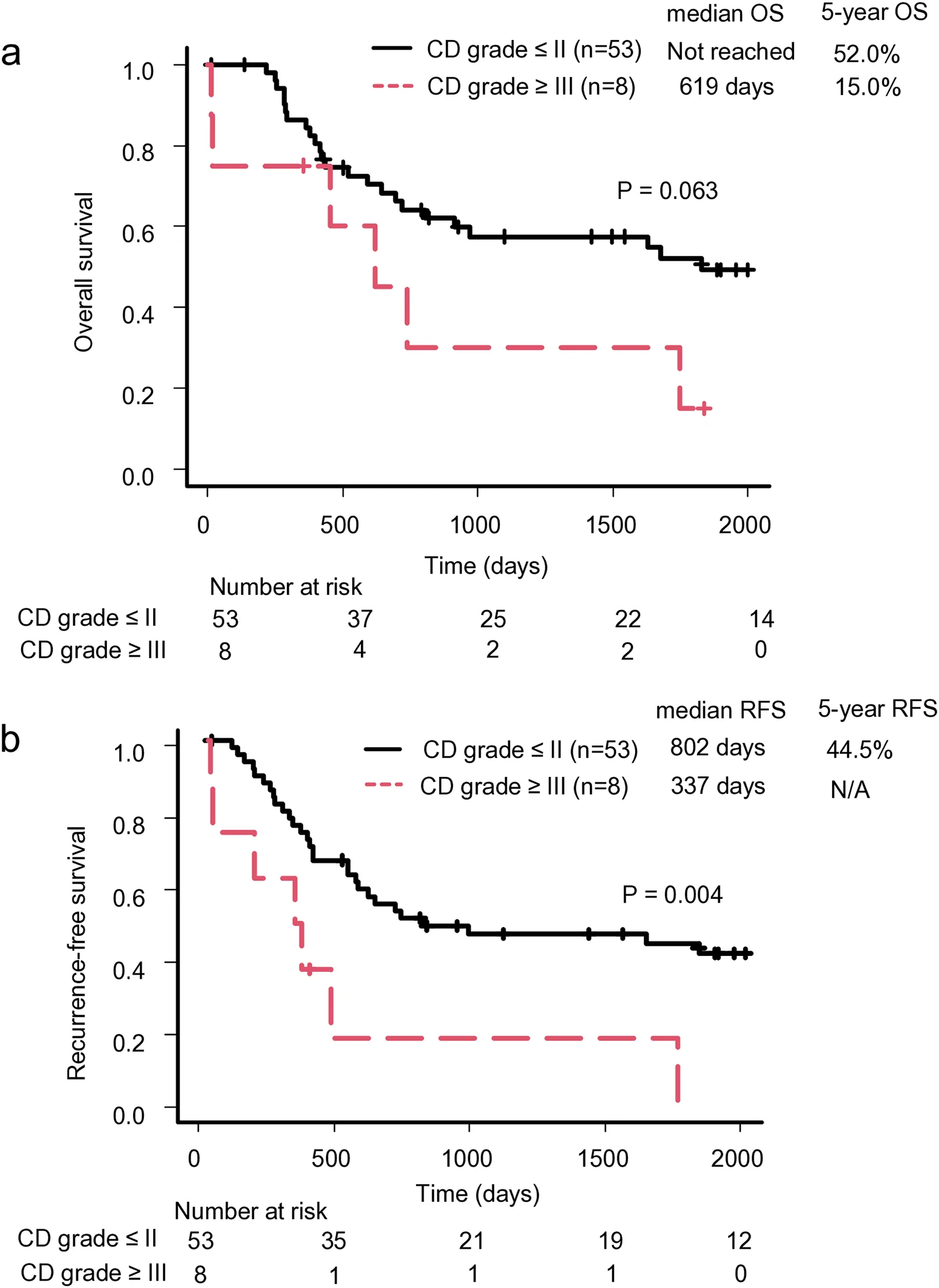
Kaplan-Meier survival analysis. a. OS estimates based on the CD grade ≥ III or ≤ II: The 5-year OS rate was 52.0% in the no or minor complication group (median OS: not reached) and 15.0% in the major complication group (median OS: 619 days). Although the difference was not statistically significant, there was a trend toward worse OS in the major complication group (P = 0.063). b. RFS estimates based on the CD grade ≥ III or ≤ II: The 5-year RFS rate was 44.5% in the no or minor complication group (median RFS: 337 days) and could not be calculated in the major complication group, as no patients remained event-free until 5 years after the operation (median RFS: 802 days). RFS was significantly worse in the major complication group (P = 0.004).

Analysis of RFS revealed significantly poorer outcomes in the major complication group compared with the no or minor complication group (P = 0.004) (Fig 1b).

### Univariate analysis

Univariate Cox regression analysis revealed that macroscopic type 4 (HR, 5.51; 95% CI, 2.25–13.49; P < 0.001), histological diffuse type (HR, 2.40; 95% CI, 1.19–4.84; P = 0.015), total gastrectomy (TG) (HR, 2.44; 95% CI, 1.19–4.97; P = 0.015), and estimated blood loss (per 100-mL increase) (HR, 1.05; 95% CI, 1.01–1.10; P = 0.027) were significantly associated with worse OS. were significantly associated with worse OS. Although not statistically significant, pT3 or deeper (HR, 2.31; 95% CI, 0.95–5.64; P = 0.066), pStage II or higher (HR, 2.83; 95% CI, 0.86–9.34; P = 0.087), and postoperative complications of CD grade ≥ III (HR, 2.23; 95% CI, 0.93–5.62; P = 0.071) showed a trend toward an association with worse OS (Table 2).

**Table 2:** Univariate survival analysis according to clinicopathological factors and postoperative complications.

Variable	HR (95% CI)	p-value
Sex (Male vs Female)	1.39 (0.67–2.89)	0.379
Respiratory disease (presence vs absence)	1.61 (0.62–4.21)	0.328
pT (3,4 vs 1,2)	2.31 (0.95–5.64)	0.066
pN (1–3 vs 0)	1.68 (0.79–3.56)	0.179
pStage (≥III vs ≤II)	1.49 (0.74–3.02)	0.265
cStage (≥III vs ≤II)	1.49 (0.74–3.02)	0.265
Macroscopic type (Type4 vs others)	5.51 (2.25–13.49)	< 0.001
Histological type (diffuse vs intestinal)	2.40 (1.19–4.84)	0.015
PNI (per 1-point decrease)	1.01 (0.94–1.07)	0.890
Operative procedure (TG vs DG)	2.44 (1.19–4.97)	0.015
Estimated blood loss (per 100-mL increase)	1.05 (1.01–1.10)	0.027
Operative time (per 30-min increase)	1.04 (0.92–1.17)	0.572
Postoperative complications (CD grade≥ III vs CD grade≤ II)	2.23 (0.93–5.62)	0.071

HR, hazard ratio; CI, confidence interval.

### Multivariate analysis

A multivariate Cox regression analysis using postoperative complications and clinically relevant pStage showed that postoperative complications of CD grade ≥ III was significantly associated with worse OS (HR, 2.51; 95% CI, 1.01–6.24; P = 0.049), whereas pStage was not significantly associated with OS (HR, 1.62; 95% CI, 0.79–3.31; P = 0.187) (Table 3-a).

**Table 3:** Multivariate survival analysis.

Variable	HR (95% CI)	p-value
**a**		
pStage (≥III vs ≤ II)	1.62 (0.79-3.31)	0.187
Postoperative complications (CD grade≥ III vs CD grade≤ II)	2.51 (1.01-6.24)	0.049
**b**		
pStage (≥III vs ≤ II)	1.16 (0.51-2.64)	0.727
Postoperative complications (CD grade≥ III vs CD grade≤ II)	2.89 (1.04-8.03)	0.042

a. Multivariate survival analysis including pStage and postoperative complications (all patients)

b. Multivariate survival analysis including pStage and postoperative complications in the subgroup excluding macroscopic type 4

### Subgroup analysis (excluding macroscopic type 4)

Since macroscopic type 4 differs substantially in clinicopathological features and prognosis from other macroscopic types, we conducted subgroup analysis excluding type 4. In the subgroup excluding patients with macroscopic type 4 tumors (*n* = 53), 46 patients were categorized into the no or minor complication group and 7 into the major complication group. The 5-year OS rate was 59.2% in the no or minor complication group (median OS: not reached) and 17.9% in the major complication group (median OS: 737 days), showing significantly worse OS in the major complication group (P = 0.036). (Fig 2).

**Fig 2:**
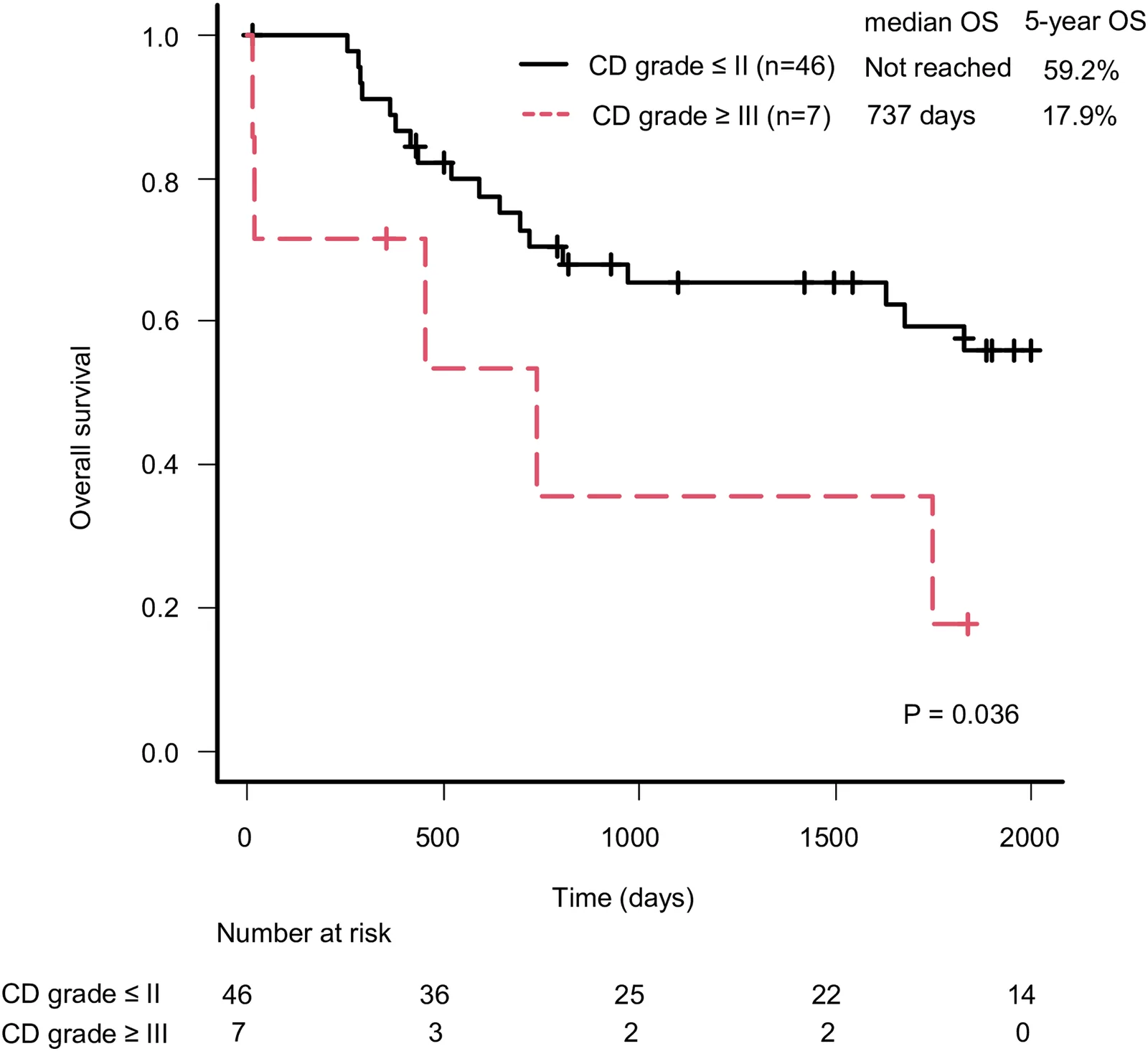
OS estimates based on the CD grade ≥ III or ≤ II in the subgroup excluding macroscopic type 4. The 5-year OS rate was 59.2% in the no or minor complication group (median OS: not reached) and 17.9% in the major complication group (median OS: 737 days) (P = 0.036).

In the univariate Cox regression analysis, postoperative complications of CD grade ≥ III was the only factor significantly associated with worse OS (HR, 2.78; 95% CI, 1.03–7.51; P = 0.044). Although not statistically significant, histological diffuse type (HR, 2.13; 95% CI, 0.96–4.73; P = 0.062) and total gastrectomy (TG) (HR, 2.06; 95% CI, 0.93–4.53; P = 0.074) showed a trend toward worse OS (Table 4).

**Table 4:** Univariate survival analysis according to clinicopathological factors and postoperative complications in the subgroup excluding macroscopic type 4.

Variable	HR (95% CI)	p-value
Sex (Male vs Female)	1.31 (0.58–2.96)	0.522
Respiratory disease (presence vs absence)	1.49 (0.44–5.02)	0.516
pT (3,4 vs 1,2)	1.86 (0.74–4.67)	0.188
pN (1–3 vs 0)	1.25 (0.56–2.79)	0.591
pStage (≥III vs ≤II)	1.01 (0.46–2.25)	0.977
cStage (≥III vs ≤II)	1.18 (0.47–2.98)	0.720
Histological type (diffuse vs intestinal)	2.13 (0.96–4.73)	0.062
PNI (per 1-point decrease)	1.02 (0.95–1.11)	0.577
Operative procedure (TG vs DG)	2.06 (0.93–4.53)	0.074
Estimated blood loss (per 100-mL increase)	1.03 (0.94–1.13)	0.508
Operative time (per 30-min increase)	1.03 (0.90–1.19)	0.653
Postoperative complications (CD grade≥ III vs CD grade≤ II)	2.78 (1.03–7.51)	0.044

A multivariate Cox regression analysis using postoperative complications and clinically relevant pStage was performed, similar to the analysis in the overall cohort. In the subgroup excluding macroscopic type 4, postoperative complications of CD grade ≥ III was significantly associated with worse OS (HR, 2.89; 95% CI, 1.04–8.03; P = 0.042), whereas pStage was not significantly associated with OS (HR, 1.16; 95% CI, 0.51–2.64; P = 0.727) (Table 3-b).

### Bootstrap internal validation

Bootstrap internal validation was performed to assess potential overfitting of the multivariable Cox regression models. The optimism-corrected slopes were 0.8459 for the overall cohort and 0.8234 for the subgroup excluding patients with macroscopic type 4 tumors, suggesting no substantial overfitting.

## Discussion

In this study, among elderly gastric cancer patients aged ≥80 years, the major complication group tended to have worse OS than the no or minor complication group. Furthermore, postoperative complications Were independently associated with poorer OS, even after adjustment for pStage. This tendency became more significant in the subgroup analysis excluding macroscopic type 4. Seo et al. [10] compared D1 and D2 lymph node dissection in elderly gastric cancer patients aged ≥80 years and reported that postoperative complications of CD grade ≥III independently predicted worse OS. To the best of our knowledge, this is the first study to focus exclusively on patients aged ≥80 years who underwent D2 lymph node dissection. Our findings are consistent with Seo’s previous report and suggested that major postoperative complications may be associated with poorer survival outcomes in elderly gastric cancer patients undergoing D2 lymph node dissection.

A comparison of baseline characteristics between the no or minor complication group and the major complication group showed no significant differences. Although previous reports have identified low albumin [25] levels, male sex [25,26], total gastrectomy [25], and excessive intraoperative blood loss [27] as risk factors for postoperative complications, no such differences were observed in this study. While the lack of significant differences in baseline characteristics may be partly due to potential selection bias, the exact reason remains unknown. Nevertheless, the lack of significant differences suggests that the groups were comparable, allowing for a valid assessment of the association between postoperative complications and survival outcomes.

The results of the subgroup analysis indicate that, particularly among elderly gastric cancer patients excluding macroscopic type 4, postoperative complications have a strong impact on OS, highlighting the importance of preventing postoperative complications in these patients. Macroscopic type 4 gastric cancer is characterized by a higher proportion of poorly differentiated tumors, more frequent lymph node and peritoneal metastases, greater serosal and lymphatic invasion, and a markedly poorer prognosis than other macroscopic types [28]. Notably, in previous Japan Clinical Oncology Group (JCOG) clinical trials, type 4 gastric cancers have been considered together with large type 3 gastric cancers as a subgroup distinct from other macroscopic types [29]. Therefore, macroscopic type 4 cases, which have an extremely poor prognosis regardless of complications, were excluded from this analysis. To clarify the cause of the poor prognosis of type 4 cases in our cohort, particularly whether it was attributable to perioperative mortality or aggressive disease progression, we reviewed the causes of death among patients with macroscopic type 4 gastric cancer. All deaths in these patients were attributable to cancer-related causes, and no perioperative deaths were observed (S3 Table). These findings suggest that excluding macroscopic type 4 cases may have allowed a clearer evaluation of the association between postoperative complications and OS; however, the underlying reasons for this difference remain unclear.

This study showed that postoperative complications were independently associated with poorer OS, even after adjustment for pStage, in elderly patients. This association may be partly explained by non-cancer-related factors. In fact, the proportion of non-cancer-related deaths was significantly higher in the major complication group than in the no or minor complication group (50.0% vs. 13.1%, P = 0.041). In the major complication group, two patients died directly from postoperative complications (CD grade V), which may have contributed to the worse OS observed in this group. However, even after excluding these postoperative deaths, two of eight patients (25%) died from non-cancer-related causes, one due to pneumonia and the other due to natural causes. (S2 Table). These findings suggest that the impact of major complications on survival cannot be explained solely by immediate postoperative mortality. The vulnerability of elderly patients may partly contribute to these findings; however, physiological reserve was not directly evaluated in this study. Future studies incorporating assessments of physiological function and geriatric status are warranted to better understand the mechanisms underlying these associations.

Furthermore, the association between postoperative complications and poorer OS may also involve cancer-related mechanisms. In this study, RFS was significantly worse in the major complication group. Tokunaga et al. [14] reported that in gastric cancer patients aged 20–80 years, postoperative complications of CD grade ≥ III significantly predicted poorer RFS, and a similar trend was observed in this study limited to patients aged ≥80 years. Previous studies involving gastric cancer patients across all age groups have reported that postoperative infectious complications can induce immunosuppression, which may subsequently lead to cancer recurrence and poor prognosis [30,31]. Therefore, we additionally evaluated the patterns of recurrence according to postoperative complication severity (S4 Table). No locoregional recurrence was observed in either group. The frequencies of peritoneal recurrence (25.0% vs. 24.5%) and lymph node recurrence (12.5% vs. 13.2%) were similar between the major complication group and the no or minor complication group. On the other hand, hematogenous recurrence was observed more frequently in the major complication group (25.0% vs. 13.2%); however, the small number of events limited further evaluation. Taken together, these findings indicate that postoperative complications may negatively influence prognosis in elderly patients through both cancer-related and non-cancer-related mechanisms. Therefore, when determining treatment strategies for elderly patients with gastric cancer, it is crucial to evaluate not only oncological factors but also physiological reserve and the risk of postoperative complications, and to provide individualized perioperative management optimized for each patient.

In addition, postoperative complications may have affected oncological outcomes by influencing the initiation and completion of postoperative adjuvant chemotherapy. Adjuvant chemotherapy was administered in a smaller proportion of patients in the major complication group than in the no or minor complication group (12.5% vs. 47.2%), and none of the patients who received adjuvant chemotherapy in the major complication group completed the planned treatment. Among the seven patients in the major complication group who did not receive adjuvant chemotherapy, three were considered ineligible because of poor PS or frailty. These findings suggest that postoperative complications may affect not only perioperative recovery but also the feasibility of postoperative adjuvant therapy, which may have contributed to the poorer OS and RFS observed in this study.

In this study, most patients underwent open gastrectomy with D2 lymph node dissection. In recent years, minimally invasive surgery (MIS), including laparoscopic and robotic-assisted gastrectomy, has become increasingly adopted for elderly patients with gastric cancer. Several previous studies have suggested that MIS is a feasible treatment option for elderly patients with gastric cancer. Kwon et al. compared MIS with open surgery specifically in patients aged ≥80 years and reported improved short-term postoperative recovery in the MIS group, while postoperative complication rates and long-term survival were comparable between the two groups [32]. In addition, Wang et al. performed a systematic review and meta-analysis demonstrating that laparoscopic gastrectomy was associated with a lower overall postoperative complication rate than open gastrectomy in elderly patients [33]. Therefore, the impact of postoperative complications on survival observed in this study may not be directly applicable to elderly patients undergoing contemporary MIS. Further studies are needed to determine whether the present findings can be generalized to patients undergoing minimally invasive gastrectomy.

This study has several limitations. First, this study was a retrospective analysis conducted at a single institution, and therefore selection bias and unmeasured confounding factors could not be completely excluded. In addition, the study period spanned more than two decades, during which surgical techniques, perioperative management, and patient selection criteria for D2 gastrectomy in octogenarians may have changed, potentially introducing additional selection bias. Second, the number of patients with severe postoperative complications was limited, which may have reduced the statistical power to detect significant differences. Subdividing this small group for subgroup analyses may have limited statistical power and increased the risk of type II errors. Because pStage was considered a clinically important prognostic factor, multivariable Cox regression analysis was performed to evaluate whether postoperative complications were independently associated with survival outcomes after adjustment for pStage. However, given the small number of patients with major postoperative complications and the limited number of events, the estimated effect of postoperative complications may have been unstable. Although bootstrap internal validation showed that both optimism-corrected slopes exceeded 0.8, suggesting no substantial overfitting, the possibility of model instability cannot be completely excluded because of the limited sample size and number of events [34]. Therefore, these findings should be interpreted with caution. Finally, postoperative complications may have affected the initiation and completion of adjuvant chemotherapy, which may in turn have been associated with survival outcomes. However, variables related to adjuvant chemotherapy were not included as covariates in the multivariable Cox regression model because of the limited number of events and the potential risk of overfitting. Therefore, the possibility that differences in adjuvant chemotherapy contributed to the observed survival outcomes cannot be completely excluded.

In conclusion, this study showed that postoperative complications of CD grade ≥ III were associated with poorer OS and RFS in elderly gastric cancer patients with D2 lymph node dissection. Future large-scale studies will be needed to further clarify their impact and optimize treatment strategies for this population.

## Supporting information

S1 Table Details of adjuvant chemotherapy according to postoperative complication group. a. Administration, completion status, and reasons for discontinuation of adjuvant chemotherapy according to postoperative complication group. b. Changes in adjuvant chemotherapy regimens during the study period.
(PDF)

S2 Table Causes and postoperative survival time of non–cancer-related deaths. (PDF)

S3 Table Causes of death among patients with macroscopic type 4 gastric cancer. (PDF)

S4 Table Patterns of recurrence according to postoperative complication status. (PDF)
