# Supplementary material for: Prognostic impact of postoperative complications among elderly patients with gastric cancer
Source: PLoS One. 2026 Sep 29;21(9):e0359100. doi: 10.1371/journal.pone.0359100 (PMC13623152; doi:10.1371/journal.pone.0359100)
Supplement: Supplementary file 1 [file pone.0359100.s001.pdf]

a

Adjuvant chemotherapy	No or minor complication group (n=53)	Major complication group (n=8)
**Received adjuvant chemotherapy**	**25 (47.2%)**	**1 (12.5%)**
Completed planned treatment	6 (11.3%)	0
Discontinued after initiation	19 (35.8%)	1 (12.5%)
Reasons for discontinuation		
Early recurrence	8 (15.1%)	1 (12.5%)
Adverse events	7 (13.2%)	0
ADL decline	3 (5.7%)	0
Patient refusal	1 (1.9%)	0
**Not received adjuvant chemotherapy**	**28 (52.8%)**	**7 (87.5%)**
**Reasons for not receiving**		
Not indicated due to early-stage disease	11 (20.8%)	0
Poor PS / frailty	7 (13.2%)	3 (37.5%)
Early recurrence	1 (1.9%)	0
Patient refusal	2 (3.8%)	1 (12.5%)
Perioperative death	0	2 (25.0%)
Unknown	7 (13.2%)	1 (12.5%)
**Regimen among patients who received chemotherapy**		
S-1	11 (20.8%)	0
UFT	8 (15.1%)	0
5-FU	1 (1.9%)	0
UFT+PSK	5 (9.4%)	1 (12.5%)

ADL, activities of daily living; S-1, tegafur/gimeracil/oteracil potassium; UFT, tegafur–uracil; 5-FU, 5-fluorouracil; PSK, protein-bound polysaccharide K.

b

Period	S-1	UFT	5-FU	UFT+PSK
**1999-2004**	0	1	1	0
**2005-2009**	2	0	7	1
**2010-2014**	3	0	0	4
**2015-2020**	6	0	0	0
