# Supplementary material for: Prognostic impact of postoperative complications among elderly patients with gastric cancer
Source: PLoS One. 2026 Sep 29;21(9):e0359100. doi: 10.1371/journal.pone.0359100 (PMC13623152; doi:10.1371/journal.pone.0359100)
Supplement: Supplementary file 2 [file pone.0359100.s002.pdf]

No.	Group (major or no/minor complication group)	Postoperative complication (CD grade)	Type of complication	Cause of death	postoperative survival time (days)
1	No/minor	None		Pneumonia	3086
2	No/minor	None		Other malignancy	719
3	No/minor	None		Unknown	2196
4	No/minor	II	Intra-abdominal abscess	Unknown	1826
5	No/minor	None		Other malignancy	1631
6	No/minor	None		Unknown	414
7	No/minor	I	Paralytic ileus	Other malignancy	520
8	No/minor	None		Natural death	696
9	Major	III	Anastomotic leakage, Pleural empyema, Pneumonia	Pneumonia	737
10	Major	V	MRSA enteritis, Sepsis	Postoperative complication (CD grade V)	18
11	Major	III	Intra-abdominal abscess	Natural death	1748
12	Major	V	Cerebral infarction	Postoperative complication (CD grade V)	10

CD, Clavien-Dindo classification; MRSA, Methicillin-resistant Staphylococcus aureus
