# Supplementary material for: Prognostic impact of postoperative complications among elderly patients with gastric cancer
Source: PLoS One. 2026 Sep 29;21(9):e0359100. doi: 10.1371/journal.pone.0359100 (PMC13623152; doi:10.1371/journal.pone.0359100)
Supplement: Supplementary file 3 [file pone.0359100.s003.pdf]

No.	Group (Major or no/minor)	Postoperative complication (CD grade)	Type of complication	Clinical management	Cause of death	Postoperative follow-up time (days)
1	No/minor	II	Intra-abdominal abscess	Antibiotic therapy	LN recurrence	397
2	No/minor	None			Peritoneal recurrence	247
3	No/minor	None			Peritoneal recurrence	282
4	No/minor	None			Peritoneal recurrence	218
5	No/minor	None			Peritoneal recurrence	422
6	No/minor	None			Peritoneal recurrence	912
7	No/minor	II	Atelectasis, Pneumonia	Antibiotic therapy	Alive at last follow-up	135
8	Major	III	Intra-abdominal abscess, MRSA enterocolitis, Paralytic ileus	Drainage, antibiotic therapy	Peritoneal recurrence	619
