# Supplementary material for: Prognostic impact of postoperative complications among elderly patients with gastric cancer
Source: PLoS One. 2026 Sep 29;21(9):e0359100. doi: 10.1371/journal.pone.0359100 (PMC13623152; doi:10.1371/journal.pone.0359100)
Supplement: Supplementary file 4 [file pone.0359100.s004.pdf]

Reccurence pattern	No or minor complication group (n=53)	Major comlication group (n=8)
No recurrence	31 (58.5%)	4 (50.0%)
Locoregional recurrence	0	0
Peritoneal recurrence	13 (24.5%)	2 (25.0%)
Hematogenous recurrence	7 (13.2%)	2 (25.0%)
Liver	3 (5.7%)	2 (25.0%)
Lung	1 (1.9%)	0
Bone	2 (3.8%)	0
Adrenal	1 (1.9%)	0
Other sites	0	1 (12.5%)
Lymph node recurrence	7 (13.2%)	1 (12.5%)

Some cases were counted more than once due to multiple recurrence patterns.
