# Supplementary material for: Prognostic impact of postoperative complications among elderly patients with gastric cancer
Source: PLoS One. 2026 Sep 29;21(9):e0359100. doi: 10.1371/journal.pone.0359100 (PMC13623152; doi:10.1371/journal.pone.0359100)
Supplement: Peer review history

# Peer review: Decision Letter 0

**DOI:** 10.1371/journal.pone.0359100.r001

**Authors:** Benjamin Benzon

10 Jul 2026

Dear Dr. Miki,

Thank you for submitting your manuscript to PLOS ONE. After careful consideration, we feel that it has merit but does not fully meet PLOS ONE’s publication criteria as it currently stands. Therefore, we invite you to submit a revised version of the manuscript that addresses the points raised during the review process.

==============================

Please submit your revised manuscript by Aug 24 2026 11:59PM. If you will need more time than this to complete your revisions, please reply to this message or contact the journal office at plosone@plos.org. When you're ready to submit your revision, log on to https://www.editorialmanager.com/pone/ and select the 'Submissions Needing Revision' folder to locate your manuscript file.

- A letter that responds to each point raised by the academic editor and reviewer(s). You should upload this letter as a separate file labeled 'Response to Reviewers'.
- A marked-up copy of your manuscript that highlights changes made to the original version. You should upload this as a separate file labeled 'Revised Manuscript with Track Changes'.
- An unmarked version of your revised paper without tracked changes. You should upload this as a separate file labeled 'Manuscript'.

If you would like to make changes to your financial disclosure, please include your updated statement in your cover letter. Guidelines for resubmitting your figure files are available below the reviewer comments at the end of this letter.

If applicable, we recommend that you deposit your laboratory protocols in protocols.io to enhance the reproducibility of your results. Protocols.io assigns your protocol its own identifier (DOI) so that it can be cited independently in the future. For instructions see: https://journals.plos.org/plosone/s/submission-guidelines#loc-laboratory-protocols. Additionally, PLOS ONE offers an option for publishing peer-reviewed Lab Protocol articles, which describe protocols hosted on protocols.io. Read more information on sharing protocols at https://plos.org/protocols?utm_medium=editorial-email&utm_source=authorletters&utm_campaign=protocols.

As the corresponding author, your ORCID iD is verified in the submission system and will appear in the published article. PLOS supports the use of ORCID, and we encourage all coauthors to register for an ORCID iD and use it as well. Please encourage your coauthors to verify their ORCID iD within the submission system before final acceptance, as unverified ORCID iDs will not appear in the published article. *Only*  the individual author can complete the verification step; PLOS staff *cannot*  verify ORCID iDs on behalf of authors.

We look forward to receiving your revised manuscript.

Kind regards,

Benjamin Benzon, Ph.D., M.D.

Academic Editor

PLOS One

Journal Requirements:

When submitting your revision, we need you to address these additional requirements.

1. Please ensure that your manuscript meets PLOS ONE's style requirements, including those for file naming. The PLOS ONE style templates can be found at

https://journals.plos.org/plosone/s/file?id=wjVg/PLOSOne_formatting_sample_main_body.pdf and

https://journals.plos.org/plosone/s/file?id=ba62/PLOSOne_formatting_sample_title_authors_affiliations.pdf

2. In the online submission form, you indicated that your data is available only on request from a third party. Please note that your Data Availability Statement is currently missing contact details for the third party, such as an email address or a link to where data requests can be made. Please update your statement with the missing information.

3. If the reviewer comments include a recommendation to cite specific previously published works, please review and evaluate these publications to determine whether they are relevant and should be cited. There is no requirement to cite these works unless the editor has indicated otherwise.

[Note: HTML markup is below. Please do not edit.]

Reviewers' comments:

Reviewer's Responses to Questions

**Comments to the Author**

1. Is the manuscript technically sound, and do the data support the conclusions?

Reviewer #1: Yes

Reviewer #2: Partly

**********

2. Has the statistical analysis been performed appropriately and rigorously?

Reviewer #1: No

Reviewer #2: Yes

**********

3. Have the authors made all data underlying the findings in their manuscript fully available?

The PLOS Data policy

Reviewer #1: Yes

Reviewer #2: Yes

**********

4. Is the manuscript presented in an intelligible fashion and written in standard English?

Reviewer #1: Yes

Reviewer #2: Yes

**********

Reviewer #1: Manuscript Number: PONE-D-26-13236

Manuscript Title: Prognostic impact of postoperative complications among elderly patients with gastric cancer

The manuscript investigates a clinically pertinent and timely issue: the prognostic impact of postoperative complications following gastrectomy with D2 lymph node dissection in an elderly cohort (≥80 years old). Utilizing a retrospective approach, the authors demonstrate that severe postoperative complications independently predict poorer overall survival (OS) and recurrence-free survival (RFS), particularly after excluding macroscopic type 4 gastric tumors. While the study offers valuable insights into the perioperative optimization of octogenarians, the long study duration, small sample size, and several critical statistical and oncological gaps undermine the validity of the current conclusions. Major revisions are mandatory to address these methodological vulnerabilities before publication can be considered.

Recommendation: Major Revision

Major revisions

1. The study concludes that preventing postoperative complications is essential to improving survival outcomes in elderly patients. However, the analysis is limited to a small group of 61 patients collected over 21 years (1999–2020) at one institution. Furthermore, only eight patients experienced major complications. Subdividing this small group limits statistical power and increases the risk of type II errors. Over the 21-year study period, the physical screening criteria for selecting patients aged 80 years and older for highly invasive D2 dissections inevitably varied, introducing a significant selection bias that cannot be fully adjusted for in a single-center study. The authors must address this limitation and adjust their clinical recommendations accordingly.

2. There is a concerning statistical paradox in the survival analyses. In the univariate analysis of the overall cohort, postoperative complications did not achieve statistical significance for OS (P = 0.071, HR 2.23). However, when the variable was entered into the multivariate Cox regression model alongside pStage, it suddenly became statistically significant (P = 0.049, HR 2.51). Adjusting for a non-significant confounder (pStage, P = 0.265) in a small sample size with a limited number of events (32 deaths) can create artifactual significance due to overfitting. The authors should consult a qualified medical statistician to verify this model. They should also consider providing a step-by-step justification or alternative propensity-score adjustments to prove that this finding is robust and not a statistical artifact.

3. The inclusion period spans more than two decades (1999–2020). Over this timeframe, standard-of-care surgical techniques, perioperative management, and, crucially, postoperative adjuvant chemotherapy regimens have changed fundamentally. One major confounding factor is whether patients who experienced severe complications were unable to receive or complete adjuvant chemotherapy. Table 1 shows that only 12.5% (one out of eight) of patients in the major complication group received adjuvant therapy, compared to 47.2% of patients in the minor group. The authors must provide explicit data on the chemotherapy regimens used in different eras, treatment completion rates, and reasons for discontinuation. Complete or incomplete delivery of chemotherapy is a significant independent driver of overall survival (OS) and relapse-free survival (RFS) that must be accounted for.

4. In the subgroup analysis excluding Type 4 tumors, the impact of complications on OS is stronger (P = 0.036). However, the mortality rate for type 4 patients within the first year post-surgery is close to 50%. Does this extremely high early mortality rate reflect direct perioperative or surgical mortality, or is it driven solely by hyper-aggressive disease recurrence? If these were perioperative deaths, they would directly overlap with the major complication window. The authors must explicitly state the exact causes of death for these patients with Type 4 tumors. Furthermore, they must provide a detailed descriptive breakdown of the specific types of complications encountered (e.g., anastomotic leak, pneumonia, and cardiovascular events) and their respective clinical management pathways.

5. The abstract and results state that recurrence-free survival (RFS) was significantly worse in the major complication group (P = 0.004). However, the manuscript fails to elaborate on how these patients experienced recurrence. Recent oncological literature suggests that severe local infections can promote distinct recurrence patterns, such as peritoneal dissemination versus hematogenous recurrence, due to systemic inflammatory responses and immunosuppression. To provide a complete oncological audit, the authors should report and analyze the specific modes of recurrence (locoregional, peritoneal, or distant) stratified by complication group.

Minor comments:

1. Documentation of Non-Cancer-Related Deaths: In the Discussion section, the authors note that two out of eight patients in the Major Complication Group died of non-cancer-related causes after excluding CD Grade V cases. They refer to "S1 Table," but this supplementary data table is not fully visible or detailed in the main text. Please ensure that all primary details regarding these specific noncancer deaths are transparent.

2. Table 1 shows that 92.5% of patients in the minor group and 100% of patients in the major group underwent open laparotomy. Since laparoscopic and robotic gastrectomies are standard practices in 2026, the discussion correctly identifies open laparotomy as a limitation. However, the text should emphasize that open D2 gastrectomy in octogenarians is more invasive than modern minimally invasive procedures.

Reviewer #2: This study addresses an important clinical question regarding the impact of major postoperative complications on the long-term prognosis of elderly patients with gastric cancer. However, several methodological and interpretative issues should be addressed before the conclusions can be considered adequately supported by the data.

First, the patient selection process requires greater transparency. Although the inclusion criteria are generally described, the exclusion criteria are not clearly presented. In particular, it remains unclear whether patients who received neoadjuvant therapy were excluded according to the study design or whether no such patients were present during the study period. Clear reporting of both the inclusion and exclusion criteria would improve the reproducibility and interpretability of the study.

Second, the rationale for dichotomizing continuous variables such as operative time, blood loss, and PNI using their median values should be clarified. Unless there is a specific clinical or methodological justification, analyzing these variables as continuous covariates and reporting hazard ratios using clinically meaningful units(e.g., per 100 mL increase in blood loss or per 30-minute increase in operative time) may preserve more information and improve the generalizability and clinical interpretability of the findings.

Third, although multivariable Cox regression was performed, the major complication group consisted of only eight patients. While the total number of outcome events may permit multivariable analysis, the estimated effect of major postoperative complications is likely to be unstable because it is based on a very small subgroup. Therefore, the results should be interpreted with appropriate caution.

More importantly, several interpretations in the Discussion appear to extend beyond what can be directly supported by the presented data. For example, the conclusion that macroscopic type 4 tumors masked the prognostic impact of postoperative complications is not sufficiently supported, particularly because the baseline distribution of macroscopic type 4 tumors did not significantly differ between the groups. Similarly, the proposed mechanism involving reduced physiological reserve remains speculative, as it was not directly evaluated in this study.

Furthermore, according to the supplementary data, two of the four non-cancer-related deaths in the major complication group were directly attributable to postoperative complications (Clavien–Dindo grade V). Consequently, the observed difference in overall survival may partly reflect postoperative mortality rather than a long-term prognostic effect of postoperative complications. This distinction should be clearly described in the Results and discussed more explicitly.

Finally, although the limitations section appropriately acknowledges the small sample size, the conclusions remain stronger than the data appear to support. Statements such as "postoperative complications predicted worse OS and RFS" imply a level of evidence that is difficult to justify in a retrospective single-center study with only eight patients in the major complication group. The conclusions should therefore be revised to emphasize an association rather than a predictive or causal relationship, and the findings should be interpreted more cautiously.

Overall, this study investigates an important clinical issue; however, clarification of the study population, more transparent presentation of the results, and a more cautious interpretation that better reflects the limitations of the data would substantially strengthen the manuscript.

**********

what does this mean?). If published, this will include your full peer review and any attached files.

If you choose “no”, your identity will remain anonymous but your review may still be made public.

**Do you want your identity to be public for this peer review?**  For information about this choice, including consent withdrawal, please see our Privacy Policy

Reviewer #1: **Yes:**  Tomoyuki Abe

Reviewer #2: No

**********

[NOTE: If reviewer comments were submitted as an attachment file, they will be attached to this email and accessible via the submission site. Please log into your account, locate the manuscript record, and check for the action link "View Attachments". If this link does not appear, there are no attachment files.]

To ensure your figures meet our technical requirements, please review our figure guidelines: https://journals.plos.org/plosone/s/figures

You may also use PLOS’s free figure tool, NAAS, to help you prepare publication quality figures: https://journals.plos.org/plosone/s/figures#loc-tools-for-figure-preparation.

NAAS will assess whether your figures meet our technical requirements by comparing each figure against our figure specifications.

# Author comment: Author response to Decision Letter 1

**DOI:** 10.1371/journal.pone.0359100.r002

26 Aug 2026

Academic Editor

PLOS One

Dear Professor Benjamin Benzon,

RE: PONE-D-26-13236

Prognostic impact of postoperative complications among elderly patients with gastric cancer

We greatly appreciate the opportunity to revise our manuscript entitled “Prognostic impact of postoperative complications among elderly patients with gastric cancer” (PONE-D-26-13236). We sincerely thank you for your thoughtful and constructive comments. We have carefully considered all comments and revised the manuscript accordingly. Detailed point-by-point responses are provided below, and all changes made in the revised manuscript are highlighted in the tracked-changes version.

This manuscript is not under consideration, in whole or in part, by any other journal, and all authors have approved the revised version and are aware of its contents.

We would prefer that the same reviewers review our paper.

We hope you will seriously consider this report for publication in PLOS One.

Sincerely,

Yuichiro Miki, M.D. PhD

Department of Gastroenterological Surgery, Osaka Metropolitan University Graduate School of Medicine

1-4-3, Asahimachi, Abenoku, Osaka City, Osaka, 545-8585, Japan

Tel: +81-6-6645-3835, Fax: +81-6-6646-6450

E-mail: y_miki@omu.ac.jp

Reviewer #1

Major revisions

1. The study concludes that preventing postoperative complications is essential to improving survival outcomes in elderly patients. However, the analysis is limited to a small group of 61 patients collected over 21 years (1999–2020) at one institution. Furthermore, only eight patients experienced major complications. Subdividing this small group limits statistical power and increases the risk of type II errors. Over the 21-year study period, the physical screening criteria for selecting patients aged 80 years and older for highly invasive D2 dissections inevitably varied, introducing a significant selection bias that cannot be fully adjusted for in a single-center study. The authors must address this limitation and adjust their clinical recommendations accordingly.

Thank you for pointing out this important issue.

We agree that the small sample size, limited number of patients with major complications, and potential changes in patient selection criteria during the 21-year study period represent important limitations.

Accordingly, we have revised the manuscript to avoid overinterpretation of the findings and to clarify these limitations as follows:

We have revised the Abstract section as follows:

“Multivariate analysis showed that postoperative complications of CD grade ≥ III independently predicted poorer OS”

was revised to:

“Multivariable analysis showed that postoperative complications of CD grade ≥ III were independently associated with poorer OS”

“This study showed that postoperative complications predicted worse OS and RFS even in patients aged ≥80 years with gastric cancer, and that reducing postoperative complications is crucial for achieving better survival outcomes.”

was revised to:

“This study showed that postoperative complications were associated with poorer OS and RFS even in patients aged ≥80 years with gastric cancer. Future large-scale studies are needed to further clarify this association and optimize treatment strategies for this population.”

We have revised the Discussion section as follows:

“Furthermore, postoperative complications significantly impacted OS, independent of pStage.”

was revised to:

“Furthermore, postoperative complications were independently associated with poorer OS, even after adjustment for pStage.”

“Our findings are consistent with Seo’s previous report and underscore the importance of preventing postoperative complications in elderly gastric cancer patients undergoing D2 lymph node dissection.”

was revised to:

“Our findings are consistent with Seo’s previous report and suggest that major postoperative complications may be associated with poorer survival outcomes in elderly gastric cancer patients undergoing D2 lymph node dissection.”

“This study showed that postoperative complications significantly impacted OS independent of pStage, in elderly patients.”

was revised to:

“This study showed that postoperative complications were independently associated with poorer OS, even after adjustment for pStage, in elderly patients.”

“In conclusion, this study showed that postoperative complications of CD grade ≥ III predicted worse OS and RFS”

was revised to:

“In conclusion, this study showed that postoperative complications of CD grade ≥ III were associated with poorer OS and RFS”

We added the following statement to the Discussion section:

“In addition, the study period spanned more than two decades, during which surgical techniques, perioperative management, and patient selection criteria for D2 gastrectomy in octogenarians may have changed, potentially introducing additional selection bias.”

“Subdividing this small group for subgroup analyses may have limited statistical power and increased the risk of type II errors.”

2. There is a concerning statistical paradox in the survival analyses. In the univariate analysis of the overall cohort, postoperative complications did not achieve statistical significance for OS (P = 0.071, HR 2.23). However, when the variable was entered into the multivariate Cox regression model alongside pStage, it suddenly became statistically significant (P = 0.049, HR 2.51). Adjusting for a non-significant confounder (pStage, P = 0.265) in a small sample size with a limited number of events (32 deaths) can create artifactual significance due to overfitting. The authors should consult a qualified medical statistician to verify this model. They should also consider providing a step-by-step justification or alternative propensity-score adjustments to prove that this finding is robust and not a statistical artifact.

Thank you for your valuable and insightful comment.

We appreciate your concern regarding the potential instability of the multivariable Cox regression model, particularly given the limited sample size, number of events, and number of patients with major postoperative complications.

We have clarified the rationale for selecting pStage as a covariate in the multivariable model. pStage was not selected based on its statistical significance in the univariate analysis, but rather based on its clinical relevance as an established prognostic factor in gastric cancer and its potential role as a confounder when evaluating the association between postoperative complications and OS.

Given the limited number of events (32 deaths), the number of covariates included in the multivariable model was restricted to minimize the risk of overfitting. Therefore, the multivariable analysis was performed using two covariates: pStage and postoperative complications.

Furthermore, we consulted medical statisticians, who performed bootstrap internal validation to evaluate potential overfitting of the multivariable Cox regression models. Potential overfitting was assessed using 2,000 bootstrap resamples with the validate function in the rms package, and optimism-corrected slopes were calculated. The optimism-corrected slopes were 0.8459 for the overall cohort and 0.8234 for the subgroup excluding patients with macroscopic type 4 tumors. Both slopes exceeded 0.8, suggesting no substantial overfitting.

However, we acknowledge that the limited number of patients with major postoperative complications represents an important limitation. Therefore, we have revised the Discussion section to clarify this limitation and to avoid overinterpretation of the findings.

We revised the Materials and Methods section as follows:

“Given the limited number of events (32 deaths), the number of variables that could be included in the multivariate analysis was restricted. Therefore, the multivariate analysis was performed using two variables: pStage and postoperative complications. Although pStage was not significant in the univariate analysis, it was included in the model as a clinically important prognostic factor to adjust for potential confounding when evaluating the effect of postoperative complications on OS.”

was revised to:

“Given the limited number of events (32 deaths), the number of variables that could be included in the multivariate analysis was restricted to avoid overfitting. Therefore, the multivariate analysis was performed using two variables: pStage and postoperative complications. pStage was included because it is an established prognostic factor in gastric cancer and was considered clinically important for adjusting for potential confounding when evaluating the association between postoperative complications and OS.”

We added the following statement to the Materials and Methods section:

“To assess potential overfitting, internal validation was performed using 2,000 bootstrap resamples with the validate function in the rms package, and optimism-corrected slopes were calculated [24].”

We added the following statement to the Results section:

“Bootstrap Internal Validation

Bootstrap internal validation was performed to assess potential overfitting of the multivariable Cox regression models. The optimism-corrected slopes were 0.8459 for the overall cohort and 0.8234 for the subgroup excluding patients with macroscopic type 4 tumors, suggesting no substantial overfitting.”

We added the following statement to the Discussion section:

“Because pStage was considered a clinically important prognostic factor, multivariable Cox regression analysis was performed to evaluate whether postoperative complications were independently associated with survival outcomes after adjustment for pStage. However, given the small number of patients with major postoperative complications and the limited number of events, the estimated effect of postoperative complications may have been unstable. Although bootstrap internal validation showed that both optimism-corrected slopes exceeded 0.8, suggesting no substantial overfitting, the possibility of model instability cannot be completely excluded because of the limited sample size and number of events [34]. Therefore, these findings should be interpreted with caution.”

In addition, the corresponding references have been added as References [24] and [34].

3. The inclusion period spans more than two decades (1999–2020). Over this timeframe, standard-of-care surgical techniques, perioperative management, and, crucially, postoperative adjuvant chemotherapy regimens have changed fundamentally. One major confounding factor is whether patients who experienced severe complications were unable to receive or complete adjuvant chemotherapy. Table 1 shows that only 12.5% (one out of eight) of patients in the major complication group received adjuvant therapy, compared to 47.2% of patients in the minor group. The authors must provide explicit data on the chemotherapy regimens used in different eras, treatment completion rates, and reasons for discontinuation. Complete or incomplete delivery of chemotherapy is a significant independent driver of overall survival (OS) and relapse-free survival (RFS) that must be accounted for.

Thank you for your valuable comment.

We agree that postoperative adjuvant chemotherapy is an important potential confounding factor, as severe postoperative complications may influence both the initiation and completion of adjuvant treatment. We have added detailed information regarding adjuvant chemotherapy administration, completion status, and reasons for discontinuation in Supplementary Table S1a, and summarized changes in chemotherapy regimens over the study period in Supplementary Table S1b.

We have also revised the manuscript as follows:

We have added the following statement to the Results section:

“Although the difference was not statistically significant, adjuvant chemotherapy was administered in a higher proportion of patients in the no or minor complication group than in the major complication group (47.2% vs. 12.5%). Among patients who received adjuvant chemotherapy, none in the major complication group completed the planned treatment (0/1), whereas 24.0% (6/25) completed treatment in the no or minor complication group (S1a Table). Changes in adjuvant chemotherapy regimens during the study period are summarized in Supplementary Table S1b.”

We have added the following statement to the Discussion section:

“In addition, postoperative complications may have affected oncological outcomes by influencing the initiation and completion of postoperative adjuvant chemotherapy. Adjuvant chemotherapy was administered in a smaller proportion of patients in the major complication group than in the no or minor complication group (12.5% vs. 47.2%), and none of the patients who received adjuvant chemotherapy in the major complication group completed the planned treatment. Among the seven patients in the major complication group who did not receive adjuvant chemotherapy, three were considered ineligible because of poor PS or frailty. These findings suggest that postoperative complications may affect not only perioperative recovery but also the feasibility of postoperative adjuvant therapy, which may have contributed to the poorer OS and RFS observed in this study.”

We have revised the Discussion section as follows:

“Finally, the administration of postoperative chemotherapy was left to the discretion of the attending physicians, which may have introduced variability in treatment strategies.

was revised to:

“Finally, postoperative complications may have affected the initiation and completion of adjuvant chemotherapy, which may in turn have been associated with survival outcomes. However, variables related to adjuvant chemotherapy were not included as covariates in the multivariable Cox regression model because of the limited number of events and the potential risk of overfitting. Therefore, the possibility that differences in adjuvant chemotherapy contributed to the observed survival outcomes cannot be completely excluded.”

4. In the subgroup analysis excluding Type 4 tumors, the impact of complications on OS is stronger (P = 0.036). However, the mortality rate for type 4 patients within the first year post-surgery is close to 50%. Does this extremely high early mortality rate reflect direct perioperative or surgical mortality, or is it driven solely by hyper-aggressive disease recurrence? If these were perioperative deaths, they would directly overlap with the major complication window. The authors must explicitly state the exact causes of death for these patients with Type 4 tumors. Furthermore, they must provide a detailed descriptive breakdown of the specific types of complications encountered (e.g., anastomotic leak, pneumonia, and cardiovascular events) and their respective clinical management pathways.

Thank you for your valuable comment.

We agree that distinguishing perioperative mortality from cancer-related mortality is important for interpreting the prognostic impact of postoperative complications, particularly in patients with macroscopic type 4 gastric cancer. We therefore reviewed the causes of death and postoperative complications in these patients and have added the relevant information in S3 Table.

We have also revised the manuscript as follows:

We have revised the Discussion section as follows:

“As a result, the effect of postoperative complications on OS was relatively more pronounced among the remaining patients.”

was revised to:

“To clarify the cause of the poor prognosis of type 4 cases in our cohort, particularly whether it was attributable to perioperative mortality or aggressive disease progression, we reviewed the causes of death among patients with macroscopic type 4 gastric cancer. All deaths in these patients were attributable to cancer-related causes, and no perioperative deaths were observed (S3 Table).”

5. The abstract and results state that recurrence-free survival (RFS) was significantly worse in the major complication group (P = 0.004). However, the manuscript fails to elaborate on how these patients experienced recurrence. Recent oncological literature suggests that severe local infections can promote distinct recurrence patterns, such as peritoneal dissemination versus hematogenous recurrence, due to systemic inflammatory responses and immunosuppression. To provide a complete oncological audit, the authors should report and analyze the specific modes of recurrence (locoregional, peritoneal, or distant) stratified by complication group.

Thank you for your valuable comment.

We agree that evaluating recurrence patterns is important to better understand the potential association between postoperative complications and oncological outcomes. We therefore analyzed the patterns of recurrence according to complication severity and have added the results in S4 Table.

We have also revised the manuscript as follows:

We added the following statement to the Discussion section:

“Therefore, we additionally evaluated the patterns of recurrence according to postoperative complication severity (S4 Table). No locoregional recurrence was observed in either group. The frequencies of peritoneal recurrence (25.0% vs. 24.5%) and lymph node recurrence (12.5% vs. 13.2%) were similar between the major complication group and the no or minor complication group. On the other hand, hematogenous recurrence was observed more frequently in the major complication group (25.0% vs. 13.2%); however, the small number of events limited further evaluation.”

Minor comments:

1. Documentation of Non-Cancer-Related Deaths: In the Discussion section, the authors note that two out of eight patients in the Major Complication Group died of non-cancer-related causes after excluding CD Grade V cases. They refer to "S1 Table," but this supplementary data table is not fully visible or detailed in the main text. Please ensure that all primary details regarding these specific noncancer deaths are transparent.

Thank you for your valuable comment.

We agree that the details of non-cancer-related deaths should be clearly described in the manuscript.

Following the addition of new supplementary tables, the table reference in the Discussion has been changed from “S1 Table” to “S2 Table”.

We have also added the following statement to the Discussion section:

“, one due to pneumonia and the other due to natural causes”

2. Table 1 shows that 92.5% of patients in the minor group and 100% of patients in the major group underwent open laparotomy. Since laparoscopic and robotic gastrectomies are standard practices in 2026, the discussion correctly identifies open laparotomy as a limitation. However, the text should emphasize that open D2 gastrectomy in octogenarians is more invasive than modern minimally invasive procedures.

Thank you for your valuable comment.

We agree that open D2 gastrectomy performed during the study period may differ substantially from contemporary minimally invasive approaches, particularly in elderly patients. Accordingly, we have revised the Discussion section as follows.

We removed the following sentence:

“Third, although many patients underwent open surgery, minimally invasive techniques such as laparoscopic and robotic-assisted surgery have become increasingly common, and it remains unclear whether the present findings are applicable to these approaches.”

Instead, we added the following new paragraph to provide a more detailed discussion supported by the relevant literature:

“In this study, most patients underwent open gastrectomy with D2 lymph node dissection. In recent years, minimally invasive surgery (MIS), including laparoscopic and robotic-assisted gastrectomy, has become increasingly adopted for elderly patients with gastric cancer. Several previous studies have suggested that MIS is a feasible treatment option for elderly patients with gastric cancer. Kwon et al. compared MIS with open surgery specifically in patients aged ≥80 years and reported improved short-term postoperative recovery in the MIS group, while postoperative complication rates and long-term survival were comparable between the two groups [32]. In addition, Wang et al. performed a systematic review and meta-analysis demonstrating that laparoscopic gastrectomy was associated with a lower overall postoperative complication rate than open gastrectomy in elderly patients [33]. Therefore, the impact of postoperative complications on survival observed in this study may not be directly applicable to elderly patients undergoing contemporary MIS. Further studies are needed to determine whether the present findings can be generalized to patients undergoing minimally invasive gastrectomy.”

In addition, the corresponding references have been added as References [32] and [33].

REVIEWER #2

First, the patient selection process requires greater transparency. Although the inclusion criteria are generally described, the exclusion criteria are not clearly presented. In particular, it remains unclear whether patients who received neoadjuvant therapy were excluded according to the study design or whether no such patients were present during the study period. Clear reporting of both the inclusion and exclusion criteria would improve the reproducibility and interpretability of the study.

Thank you for your valuable comment.

We agree that clarification of the inclusion and exclusion criteria is important for improving the reproducibility and interpretability of the study.

We have revised the Materials and Methods section to clearly describe the patient selection process. Specifically, we clarified that all consecutive patients with gastric cancer who underwent gastrectomy at our institution during the study period were retrospectively identified. We also specified the eligibility and exclusion criteria used to define the study population. In addition, we defined neoadjuvant therapy as an exclusion criterion. However, no patients who met the eligibility criteria had received neoadjuvant therapy; this was clarified in the Results section.

We have revised the Materials and Methods section as follows:

“We retrospectively analyzed a cohort of elderly patients who underwent gastrectomy with D2 lymph node dissection for advanced gastric cancer at our institution between January 1, 1999, and December 31, 2020. Patients aged ≥80 years at the time of surgery were included, and data were collected from medical records. Patients with cT1 disease and those who underwent palliative surgery were excluded. A total of 61 patients met the inclusion criteria and were included in the final analysis.”

was revised to:

“We retrospectively identified all consecutive patients with gastric cancer who underwent gastrectomy at our institution between January 1, 1999, and December 31, 2020. Clinical data were collected from medical records. The eligibility and exclusion criteria were as follows:

Eligibility criteria

� Patients diagnosed with gastric cancer.

� Age ≥80 years at the time of surgery.

� Patients who underwent curative-intent surgery with D2 lymph node dissection.

Exclusion criteria

� cT1 disease.

� Patients who received neoadjuvant therapy.

Finally, 61 patients were included in the analysis.”

In addition, to avoid potential confusion regarding the patient selection process, the following statement was deleted from the Materials and Methods section:

“No patients received neoadjuvant chemotherapy.”

And we have revised the Results section as follows:

“None of the patients received any preoperative treatments, including chemotherapy or radiotherapy.”

was revised to:

“Among the patients who met the eligibility criteria, none received neoadjuvant therapy, including chemotherapy or radiotherapy.”

Second, the rationale for dichotomizing continuous variables such as operative time, blood loss, and PNI using their median values should be clarified. Unless there is a specific clinical or methodological justification, analyzing these variables as continuous covariates and reporting hazard ratios using clinically meaningful units(e.g., per 100 mL increase in blood loss or per 30-minute increase in operative time) may preserve more information and improve the generalizability and clinical interpretability of the findings.

Thank you for your valuable comment.

We agree that dichotomization of continuous variables using median values may reduce statistical power and clinical interpretability. Therefore, we reanalyzed PNI, estimated blood loss, and operative time as continuous variables rather than using median-based cutoff values.

We have revised the manuscript as follows:

We have revised Table 1 by presenting “Operative time” and “Estimated blood loss” as continuous variables rather than dichotomized variables.

We have deleted the following statement from the Materials and Methods section:

“Patients were stratified into groups using the median values of operative time (212 minutes), estimated blood loss (295 mL), and PNI (43.3) as cutoff values.”

We have added the following statement to the Materials and Methods section:

“Continuous variables, including PNI, estimated blood loss, and operative time, were analyzed as continuous covariates in the Cox proportional hazards model. Hazard ratios were calculated per 1-point decrease in PNI, per 100-mL increase in estimated blood loss, and per 30-minute increase in operative time.”

We have revised Table 2 by reanalyzing “PNI”, “Estimated blood loss”, and “Operative time” as continuous variables using clinically meaningful units (per 1-point decrease in PNI, per 100-mL increase in estimated blood loss, and per 30-minute increase in operative time) instead of dichotomizing them based on median values.

Accordingly, we have revised the Results section as follows:

“Univariate Cox regression analysis revealed that macroscopic type 4 (HR, 5.51; 95% CI, 2.25–13.49; P < 0.001), histological diffuse type (HR, 2.40; 95% CI, 1.19–4.84; P = 0.015), and total gastrectomy (TG) (HR, 2.44; 95% CI, 1.19–4.97; P = 0.015) were significantly associated with worse OS. Although not statistically significant, pT3 or deeper (HR, 2.31; 95% CI, 0.95–5.64; P = 0.066), pStage II or higher (HR, 2.83; 95% CI, 0.86–9.34; P = 0.087), intraoperative blood loss ≥295 mL (HR, 2.00; 95% CI, 0.98–4.12; P = 0.059), and postoperative complications of CD grade ≥ III (HR, 2.23; 95% CI, 0.93–5.62; P = 0.071) showed a trend toward an association with worse OS (Table 2).”

was revised to:

“Univariate Cox regression analysis revealed that macroscopic type 4 (HR, 5.51; 95% CI, 2.25–13.49; P < 0.001), histological diffuse type (HR, 2.40; 95% CI, 1.19–4.84; P = 0.015), total gastrectomy (TG) (HR, 2.44; 95% CI, 1.19–4.97; P = 0.015), and estimated blood loss (per 100-mL increase) (HR, 1.05; 95% CI, 1.01–1.10; P = 0.027) were significantly associated with worse OS. were significantly associated with worse OS. Although not statistically significant, pT3 or deeper (HR, 2.31; 95% CI, 0.95–5.64; P = 0.066), pStage II or higher (HR, 2.83; 95% CI, 0.86–9.34; P = 0.087), and postoperative complications of CD grade ≥ III (HR, 2.23; 95% CI, 0.93–5.62; P = 0.071) showed a trend toward an association with worse OS (Table 2).”

We have also revised Table 4 by reanalyzing “PNI”, “Estimated blood loss”, and “Operative time” as continuous variables using clinically meaningful units (per 1-point decrease in PNI, per 100-mL increase in estimated blood loss, and per 30-minute increase in operative time) instead of dichotomizing them based on median values.

Third, although multivariable Cox regression was performed, the major complication group consisted of only eight patients. While the total number of outcome events may permit multivariable analysis, the estimated effect of major postoperative complications is likely to be unstable because it is based on a very small subgroup. Therefore, the results should be interpreted with appropriate caution.

Thank you for your valuable comment.

We agree that the small number of patients with major postoperative complications may have resulted in unstable estimates in the multivariable Cox regression analysis.

We also consulted medical statisticians regarding the statistical analysis. Bootstrap internal validation did not suggest substantial overfitting; however, given the limited sample size and number of events, model instability cannot be completely excluded.

We have therefore revised the Discussion section to emphasize this limitation and the need for cautious interpretation of the findings.

We have revised the Discussion section as follows:

“Second, the number of patients with severe postoperative complications was limited, which may have reduced the statistical power to detect significant differences.”

was revised to:

“Second, the number of patients with severe postoperative complications was limited, which may have reduced the statistical power to detect significant differences. Subdividing this small group for subgroup analyses may have limited statistical power and increased the risk of type II errors. Because pStage was considered a clinically important prognostic factor, multivariable Cox regression analysis was performed to evaluate whether postoperative complications were independently associated with survival outcomes after adjustment for pStage. However, given the small number of patients with major postoperative complications and the limited number of events, the estimated effect of postoperative complications may have been unstable. Although bootstrap internal validation showed that both optimism-corrected slopes exceeded 0.8, suggesting no substantial overfitting, the possibility of model instability cannot be completely excluded because of the limited sample size and number of events [33]. Therefore, these findings should be interpreted with caution.”

More importantly, several interpretations in the Discussion appear to extend beyond what can be directly supported by the presented data. For example, the conclusion that macroscopic type 4 tumors masked the prognostic impact of postoperative complications is not sufficiently supported, particularly because the baseline distribution of macroscopic type 4 tumors did not significantly differ between the groups. Similarly, the proposed mechanism involving reduced physiological reserve remains speculative, as it was not directly evaluated in this study.

Thank you for your valuable comment.

We agree that some interpretations in the Discussion may have extended beyond what could be directly supported by our data.

We have revised the Discussion section to avoid overinterpretation and to clarify that these findings represent associations rather than definitive mechanisms. In addition, we have added background information regarding the clinical distinction of macroscopic type 4 and large type 3 gastric cancers in previous JCOG clinical trials to provide context for the subgroup analysis excluding macroscopic type 4 cases.

We have revised the Discussion section as follows:

“These findings suggest that, in the overall cohort, the overwhelming poor prognosis of macroscopic type 4 cases may mask the impact of postoperative complications, and that excluding these cases allows for a clearer assessment of their independent effect.”

was revised to:

“These findings suggest that excluding macroscopic type 4 cases may have allowed a clearer evaluation of the association between postoperative complications and OS; however, the underlying reasons for this difference remain unclear.”

In addition, we added the following sentence to the Discussion section:

“Notably, in previous Japan Clinical Oncology Group (JCOG) clinical trials, type 4 gastric cancers have been considered together with large type 3 gastric cancers as a subgroup distinct from other macroscopic types [29].”

We also added the corresponding reference [29] to the reference list.

We have also revised the Discussion section as follows:

“These findings suggest that reduced physiological reserve in elderly patients may render postoperative complications potentially fatal, and limited recovery capacity may directly affect survival outcomes.”

was revised to:

“The vulnerability of elderly patients may partly contribute to these findings; however, physiological reserve was not directly evaluated in this study. Future studies incorporating assessments of physiological function and geriatric status are warranted to better understand the mechanisms underlying these associations.”

Furthermore, according to the supplementary data, two of the four non-cancer-related deaths in the major complication group were directly attributable to postoperative complications (Clavien–Dindo grade V). Consequently, the observed difference in overall survival may partly reflect postoperative mortality rather than a long-term prognostic effect of postoperative complications. This distinction should be clearly described in the Results and discussed more explicitly.

Thank you for your valuable comment.

We agree that postoperative mortality due to Clavien–Dindo grade V complications should be distinguished from the potential long-term prognostic impact of postoperative complications.

We have revised the manuscript as follows:

We have added the following statement to the Results section:

“Among the non-cancer-related deaths, two patients in the major complication group died directly from postoperative complications (CD grade V) (S1 Table).”

We have revised the Discussion section as follows:

“Although the two CD grade V cases in the major complication group may have strongly influenced this result, even after excluding them, two of eight patients (25%) died from non-cancer-related causes”

was revised to:

“In the major complication group, two patients died directly from postoperative complications (CD grade V), which may have contributed to the worse OS observed in this group. However, even after excluding these postoperative deaths, two of eight patients (25%) died from non-cancer-related causes”

We have also added the following statement to the Discussion section:

“These findings suggest that the impact of major complications on survival cannot be explained solely by immediate postoperative mortality.”

Finally, although the limitations section appropriately acknowledges the small sample size, the conclusions remain stronger than the data appear to support. Statements such as "postoperative complications predicted worse OS and RFS" imply a level of evidence that is difficult to justify in a retrospective single-center study with only eight patients in the major complication group. The conclusions should therefore be revised to emphasize an association rather than a predictive or causal relationship, and the findings should be interpreted more cautiously.

Thank you for your valuable comment.

We agree that, given the retrospective single-center design and the limited number of patients with major postoperative complications, our findings should be interpreted with caution. Accordingly, we have revised the manuscript to explicitly emphasize this point in the limitations section. In addition, we have revised the conclusions throughout the manuscript by replacing predictive or causal expressions (e.g., "predicted worse OS and RFS") with more appropriate association-based wording (e.g., "were associated with poorer OS and RFS").

We have revised the manuscript as follows:

We revised the Abstract section as follows:

“Multivariate analysis showed that postoperative complications of CD grade ≥ III independently predicted poorer OS”

was revised to:

“Multivariable analysis showed that postoperative complications of CD grade ≥ III were independently associated with poorer OS”

“This study showed that postoperative complications predicted worse OS and RFS even in patients aged ≥80 years with gastric cancer, and that reducing postoperative complications is crucial for achieving better survival outcomes.”

was revised to:

“This study showed that postoperative complications were associated with poorer OS and RFS even in patients aged ≥80 years with gastric cancer. Future large-scale studies are needed to further clarify this association and optimize treatment strategies for this population.”

We revised the Discussion section as follows:

“Furthermore, postoperative complications significantly impacted OS, independent of pStage.”

was revised to:

“Furthermore, postoperative complications were independently associated with poorer OS, even after adjustment for pStage.”

“Our findings are consistent with Seo’s previous report and underscore the importance of preventing postoperative complications in elderly gastric cancer patients undergoing D2 lymph node dissection.”

was revised to:

“Our findings are consistent with Seo’s previous report and suggest that major postoperative complications may be associated with poorer survival outcomes in elderly gastric cancer patients undergoing D2 lymph node dissection.”

“This study showed that postoperative complications significantly impacted OS independent of pStage, in elderly patients.”

was revised to:

“This study showed that postoperative complications were independently associated with poorer OS, even after adjustment for pStage, in elderly patients.”

“In conclusion, this study showed that postoperative complications of CD grade ≥ III predicted worse OS and RFS”

was revised to:

“In conclusion, this study showed that postoperative complications of CD grade ≥ III were associated with poorer OS and RFS”

We have added the following statement to the Discussion section:

“Therefore, these findings should be interpreted with caution.”

Attachment Submitted filename: Response_to_reviewers.docx

# Editor report: Decision Letter 1

**DOI:** 10.1371/journal.pone.0359100.r003

**Authors:** Benjamin Benzon

9 Sep 2026

<p>Prognostic impact of postoperative complications among elderly patients with gastric cancer

PONE-D-26-13236R1

Dear Dr. Miki,

We’re pleased to inform you that your manuscript has been judged scientifically suitable for publication and will be formally accepted for publication once it meets all outstanding technical requirements.

Within one week, you’ll receive an e-mail detailing the required amendments. When these have been addressed, you’ll receive a formal acceptance letter and your manuscript will be scheduled for publication.

An invoice will be generated when your article is formally accepted. Please note, if your institution has a publishing partnership with PLOS and your article meets the relevant criteria, all or part of your publication costs will be covered. Please make sure your user information is up-to-date by logging into Editorial Manager at Editorial Manager® and clicking the ‘Update My Information' link at the top of the page. For questions related to billing, please contact billing support.

If your institution or institutions have a press office, please notify them about your upcoming paper to help maximize its impact. If they’ll be preparing press materials, please inform our press team as soon as possible -- no later than 48 hours after receiving the formal acceptance. Your manuscript will remain under strict press embargo until 2 pm Eastern Time on the date of publication. For more information, please contact onepress@plos.org.

Kind regards,

Benjamin Benzon, Ph.D., M.D.

Academic Editor

PLOS One

Additional Editor Comments (optional):

Reviewers' comments:

# Editor report: Acceptance letter

**DOI:** 10.1371/journal.pone.0359100.r004

**Authors:** Benjamin Benzon

PONE-D-26-13236R1

PLOS One

Dear Dr. Miki,

I'm pleased to inform you that your manuscript has been deemed suitable for publication in PLOS One. Congratulations! Your manuscript is now being handed over to our production team.

At this stage, our production department will prepare your paper for publication. This includes ensuring the following:

* All references, tables, and figures are properly cited

* All relevant supporting information is included in the manuscript submission,

* There are no issues that prevent the paper from being properly typeset

You will receive further instructions from the production team, including instructions on how to review your proof when it is ready. Please keep in mind that we are working through a large volume of accepted articles, so please give us a few days to review your paper and let you know the next and final steps.

Lastly, if your institution or institutions have a press office, please let them know about your upcoming paper now to help maximize its impact. If they'll be preparing press materials, please inform our press team within the next 48 hours. Your manuscript will remain under strict press embargo until 2 pm Eastern Time on the date of publication. For more information, please contact onepress@plos.org.

You will receive an invoice from PLOS for your publication fee after your manuscript has reached the completed accept phase. If you receive an email requesting payment before acceptance or for any other service, this may be a phishing scheme. Learn how to identify phishing emails and protect your accounts at https://explore.plos.org/phishing.

If we can help with anything else, please email us at customercare@plos.org.

Thank you for submitting your work to PLOS One and supporting open access.

Kind regards,

PLOS One Editorial Office Staff

on behalf of

Dr. Benjamin Benzon

Academic Editor

PLOS One
